# Microstructure and performance of rare earth element-strengthened plasma-facing tungsten material

**DOI:** 10.1038/srep32701

**Published:** 2016-09-06

**Authors:** Laima Luo, Jing Shi, Jinshan Lin, Xiang Zan, Xiaoyong Zhu, Qiu Xu, Yucheng Wu

**Affiliations:** 1 School of Materials Science and Engineering, Hefei University of Technology, Hefei 230009, People’s Republic of China; 2 National–Local Joint Engineering Research Centre of Nonferrous Metals and Processing Technology, Hefei 230009, People’s Republic of China; 3 Research Reactor Institute, Kyoto University, Osaka-fu 590-0494, Japan

## Abstract

Pure W and W-(2%, 5%, 10%) Lu alloys were manufactured via mechanical alloying for 20 h and a spark plasma sintering process at 1,873 K for 2 min. The effects of Lu doping on the microstructure and performance of W were investigated using various techniques. For irradiation performance analysis, thermal desorption spectroscopy (TDS) measurements were performed from room temperature to 1,000 K via infrared irradiation with a heating rate of 1 K/s after implantations of He^+^ and D^+^ ions. TDS measurements were conducted to investigate D retention behavior. Microhardness was dramatically enhanced, and the density initially increased and then decreased with Lu content. The D retention performance followed the same trend as the density. Second-phase particles identified as Lu_2_O_3_ particles were completely distributed over the W grain boundaries and generated an effective grain refinement. Transgranular and intergranular fracture modes were observed on the fracture surface of the sintered W-Lu samples, indicating some improvement of strength and toughness. The amount and distribution of Lu substantially affected the properties of W. Among the investigated alloy compositions, W-5%Lu exhibited the best overall performance.

Controlled thermonuclear fusion energy has been identified as one of the most promising approaches for addressing future energy problems. The International Thermonuclear Experimental Reactor (ITER) program will demonstrate the scientific validity and viability of secure fusion energy by burning deuterium (D)-tritium (T) plasma integrated with key reactor technologies^1^. W has been the focus of considerable attention and is regarded as more potentially suitable plasma-facing materials (PFMs) in fusion devices than low-Z materials^2^. W is considered a suitable PFM because of its remarkable properties, which include a high melting point, low sputtering erosion rate, and low tritium retention. However, extremely harsh environmental conditions may cause problems. The W material will be exposed to substantially high heat fluxes and to intense neutron and hydrogen isotopes and helium (He) plasma radiation during long-term operation of a fusion reactor. These circumstances will give rise to changes in the surface morphology, leading to retention of hydrogen isotopes and helium (He), blistering in W, and dramatic impairment of the thermal and mechanical performances of W. Additionally, W shows various brittleness characteristics, including embrittlement induced by low-temperature recrystallization and irradiation. Embrittlement tends to lead to cracking and failure within the W matrix under harsh conditions. Therefore, the mechanical and irradiation properties of W play a critical role in fusion reactors.

Over the past few years, researchers have attempted to partly modify the performance of W via micro-alloying and doping of dispersed hard particles to manufacture ultrafine-grained W-based materials with a stabilized microstructure^3^. Fine-grained W materials not only are strong and tough but also exhibit improved radiation resistance. Fine-grained materials have more grain boundaries that serve as effective sinks for bombarding particles and various crystal defects to diminish radiation damage^4^,^5^. Alloying is one of the most common methods used to improve the performance of W-based materials. The doping elements can diffuse and dissolve into the W matrix or act on the defects and impurities to change the W texture, thus improving its properties. Numerous attempts to manufacture modified alloyed W materials, for instance, W-V^6^,^7^, W-Ti^8^, W-Ta^9^,^10^, W-Y^11^, and W-Zr^12^ alloys, have been reported. The reinforcement mechanism of alloyed W mainly includes solution and interface strengthening. Alloying elements such as Re, Hf, Zr, and Nb help modify impurity distributions and effectively strengthen the grain boundaries of W^13^. In addition, rare-earth (RE) elements such as La and Y show strong affinities for oxygen, thus forming oxide nanoparticles such as La_2_O_3_ and Y_2_O_3_, respectively. These dispersed particles could effectively inhibit grain growth and refine the W grains^14^. In the present work, we added Lu to the W matrix to analyze the effect of an RE element on the mechanical properties and irradiation performance of W.

As was mentioned above, in the ITER and future fusion devices, the first wall material will be exposed to a high flux (10^20^ m^−2^ s^−1^ to 10^24^ m^−2^ s^−1^) of D and T fuel particles at energies ranging from the eV to the keV range^15^. The high retention of the hydrogen isotopes in the PFM would not only degrade the fueling efficiency of burning plasma but also increase the radioactive inventory after the shutdown of the device, which would pose critical safety hazards. Therefore, low D or T retention is a key parameter for estimating the applicability of a first wall material and is vital for efficiency, safety, and economic concerns^16^,^17^.

In the present study, W-Lu alloys were fabricated by mechanical alloying (MA) and spark plasma sintering (SPS) process. SPS technology was applied to control grain growth by utilizing a direct current and uniaxial high pressure to produce small samples with fine grains^18^,^19^. The microstructure and mechanical properties of the alloys were characterized. D and He irradiations were performed to investigate the change of irradiation performance Lu brings to W and how He pre-irradiation affects D retention in W.

## Experimental Procedure

### Synthesis and consolidation of W-LuH_2_ composite powder

Pure W (W012, purity >99.9%, Xiamen Golden Egret Special Alloy Co. Ltd., Fujian, China) and LuH_2_ powders (purity >99.75%, Titd Metal Materials Co. Ltd., Changsha, China) were used to fabricate W-(0, 2, 5, 10) wt.% LuH_2_ composite powders via the MA method. The particle sizes of W and LuH_2_ powders were 1.2 and 74 μm, respectively. All mixed powders were milled in a nylon ball miller for 20 h using tungsten carbide balls; the ball-to-powder weight ratio was 10:1, and the rotation rate was 400 rpm. LuH_2_ was not oxidized during the milling process due to its stability. Ethyl alcohol was used as the medium to prevent the agglomeration of the composite powders. The alloyed powders were extracted from the ball miller chamber after the alcohol was completely evaporated in an air drying oven.

**Figure 1:**
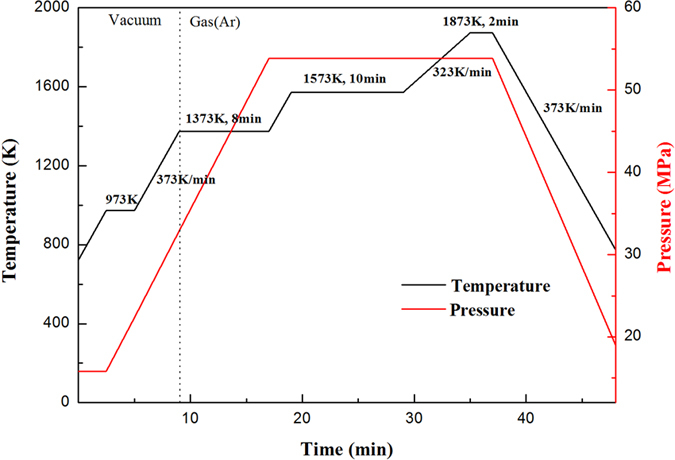
Temperature and pressure profile of the SPS process of the W and the W-Lu alloys.

The prepared pure W and W-LuH_2_ composite powders were consolidated by SPS (FCT Group, SE-607, Germany). The powders were placed in a graphite mold at 1,873 K for 2 min under a uniaxial pressure of 53.8 MPa. The specific temperature and pressure curve of the sintering process is shown in Fig. 1. The powders were heated by pulsed current to 1,573 K at a heating rate of approximately 373 K/min. LuH_2_ was expected to decompose into pure Lu and H_2_ at temperatures beyond 1,173 K. The decomposition mainly occurred under high vacuum conditions to facilitate the release of the gas from the sintering powder. The subsequent sintering and cooling processes were performed under the protection of a flowing Ar atmosphere to avoid oxidation. A dwell time of 8 min at 1,373 K was used to ensure that the residual LuH_2_ was completely decomposed. The samples were then heated to 1,573 K. After a holding time of 10 min, the samples were heated to 1,873 K at a rate of 323 K/min. Given that the melting point of Lu is 1,936 K, a maximum sintering temperature of 1,873 K was used to avoid melting during the SPS process. After 2 min at 1,873 K, the samples were cooled to room temperature at a cooling rate of approximately 373 K/min. Simultaneously, the pressure decreased uniformly. The obtained sintered compacts were disk-like components with a diameter and thickness of approximately 20 mm and 2.0 mm, respectively.

### Characterization

The actual density of each sample was measured using the Archimedes’ immersion method. The theoretical densities of W-Lu alloys were calculated from the mass fractions and theoretical densities of W and Lu, which were 19.25 and 9.84 g/cm^3^, respectively. The Vickers microhardness of the polished specimens was determined by the average of multiple measurements using an MH-3L microhardness tester with a load of 2.94 N (300 gf) maintained for 10 s.

X-ray diffraction (XRD, D/MAX2, 500 V, Japan) analysis was performed to identify the purity of the obtained powders and the phases present in the system. The particle morphology of the milled powders and the surface microstructure and fracture morphology of the sintered compacts were investigated by field-emission scanning electron microscopy (FESEM, SU8020, Japan). The composition analysis was performed using energy-dispersive spectroscopy (EDS) during the SEM analysis. The detailed microstructure was examined by transmission electron microscopy (TEM, JEM-2100 F, Japan), conducted at an acceleration voltage of 300 kV. The disk for TEM was prepared from the W-10%Lu alloy sample using a linear cutting machine and was ground with SiC abrasive paper to a thickness of approximately 50 μm. A dimpling grinder and ion-thinning system (model 691, Gatan) were used to make the disk thinner to satisfy the TEM specimen requirement.

For irradiation performance analysis, pure W and W-(2%, 5%, 10%) Lu composites were irradiated with 5 keV D^+^ ions to a fluence of 1 × 10^20^ D/m^2^ under ultra-high vacuum. One of the W-5%Lu samples was sequentially irradiated to 5 keV He^+^ and 5 keV D^+^ ions with fluences of 1 × 10^21^ He/m^2^ and 1 × 10^20^ D/m^2^, respectively, to investigate the effects of He^+^ irradiation on D retention. After these ion implantations, thermal desorption spectroscopy (TDS) measurements were performed from room temperature to 1,000 K via infrared irradiation with a heating rate of 1 K/s to investigate the D retention behavior. D_2_ molecules were detected in the TDS measurement, and the total D retention was calculated from the obtained D_2_ amount. In all cases, the samples were stored in air during the interval between the end of exposure and the beginning of the TDS measurement. The diameter and thickness of all the samples for TDS were 3 mm and 0.1 mm, respectively. Prior to the measurement, the surfaces of the samples were mechanically polished to a mirror-like finish.

## Results and Discussions

### Characterization of as-received powders

**Figure 2:**
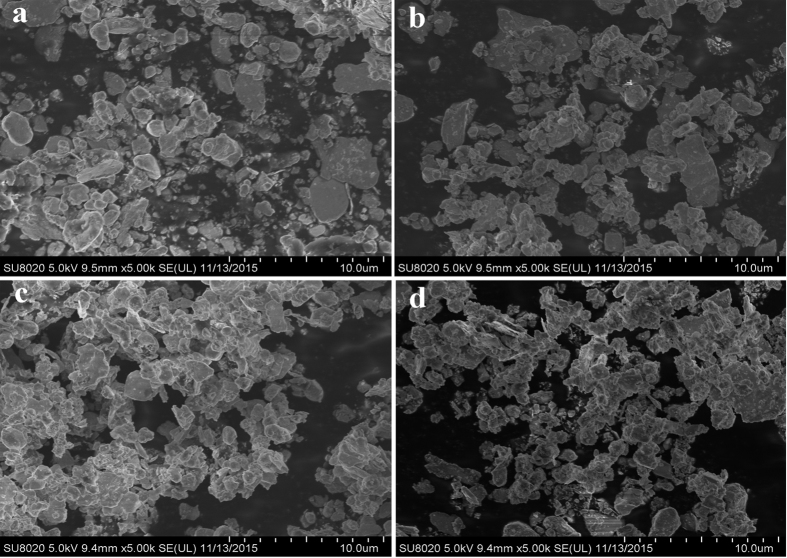
FESEM images of milled pure W and W-LuH_2_ composite powder. (**a**) pure W; (**b**) W-2%Lu; (**c**) W-5%Lu; and (**d**) W-10%Lu.

**Figure 3:**
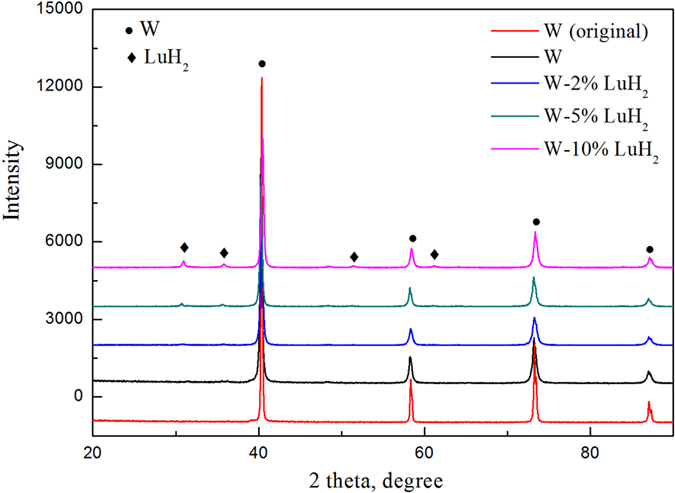
XRD patterns of original W (before milling), pure W and W-LuH_2_ composite powders with different contents of LuH_2_ after the samples were ball-milled for 20 h.

The FESEM micrographs of pure W and different W-LuH_2_ powders after MA are presented in Fig. 2. All the powders comprise large particles with irregular shapes. The powders were ground, work hardened, and fractured by fragmentation after high-energy ball milling for 20 h. The powders were refined until a single particle was approximately 1 μm. However, powder agglomeration was observed for all the powders, and the degree of agglomeration increased with increasing LuH_2_ content. This aggregation is related to the significantly increased surface energy and activity of powders prepared by MA. The constituent phases of the obtained powders and those of the original (before milling) W powder were examined via XRD; the results are shown in Fig. 3. The four main peaks are attributed to pure W (JCPDS#04-0806), whereas the relatively weak peaks are identified as LuH_2_ (JCPDS#42-0983). LuH_2_ powders were not oxidized during the ball milling process because of the good stability of LuH_2_. Compared to the diffraction pattern of the pure W powder, the patterns of the composite powders show less intense and broader peaks, indicating the occurrence of grain refinement and inner stress after ball milling.

### Characterization of sintered pure W and W-Lu alloys

**Figure 4:**
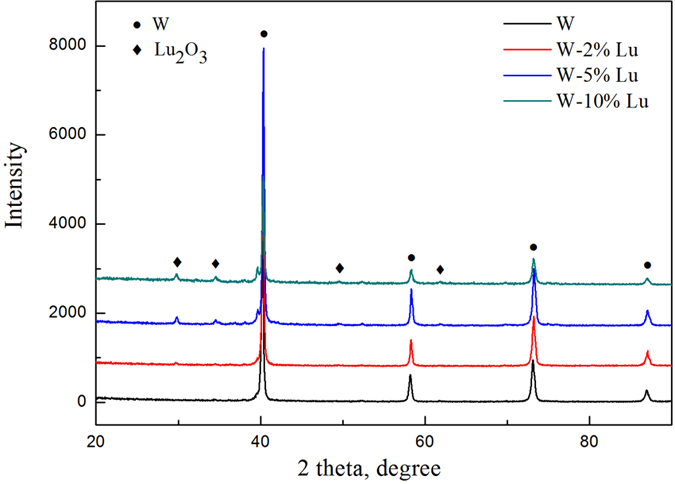
XRD pattern of sintered pure W and W-Lu samples with different contents of Lu.

**Figure 5:**
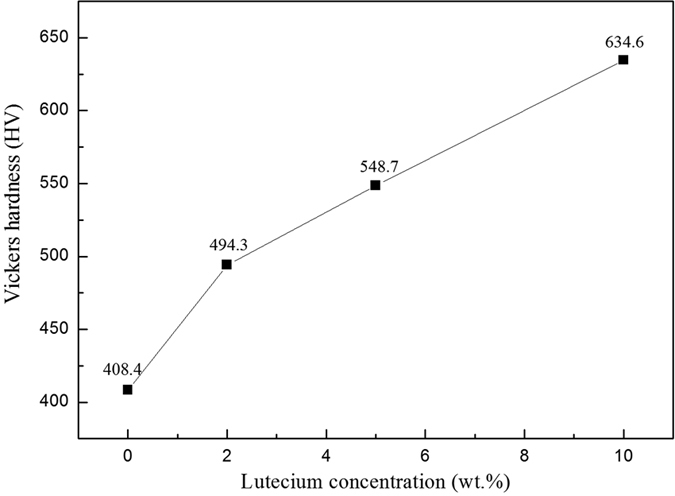
Microhardness of pure W and W-Lu alloys.

XRD analysis was also used to determine the constituent phases of the sintered samples, as shown in Fig. 4. Unlike for the as-received composite powders, the relatively weak peaks belong to the Lu_2_O_3_ phase (JCPDS#12-0728). This result indicates that the reduced Lu captured oxygen impurities in the W to form the oxide during the sintering process. The experimental densities of pure W and W-Lu alloys were measured using the Archimedes principle. Table 1 displays the densities and relative densities of the different samples. The composite powders were sintered by SPS at 1,873 K for 2 min. However, the overall relative densities were low after sintering. The highest relative density, which was obtained in the case of the W-5%Lu alloy, was only 94.7%. The low densities may be related to the substantial release of hydrogen resulting from the decomposition of LuH_2_ powders during the sintering process. As shown in Table 1, the relative densities of the samples initially increased with increasing Lu content and then slightly decreased in the case of the W-10%Lu alloy. This result indicates that an appropriate Lu content benefits densification during the sintering process. Excessive addition of Lu may hinder atom migration, and the density of Lu is relatively low compared to that of W; both of these factors may be responsible for the lower relative density of the W-10%Lu alloy. The larger amount of gas released also impeded the densification process. Figure 5 shows the variation trend of microhardness with respect to Lu addition. The hardness of W was dramatically improved by Lu, increasing from 408.4 Hv to 634.6 Hv. The dispersive phases on the W grain boundaries can refine the grains, inhibit the movement of dislocations and thus increase the hardness.

**Figure 6:**
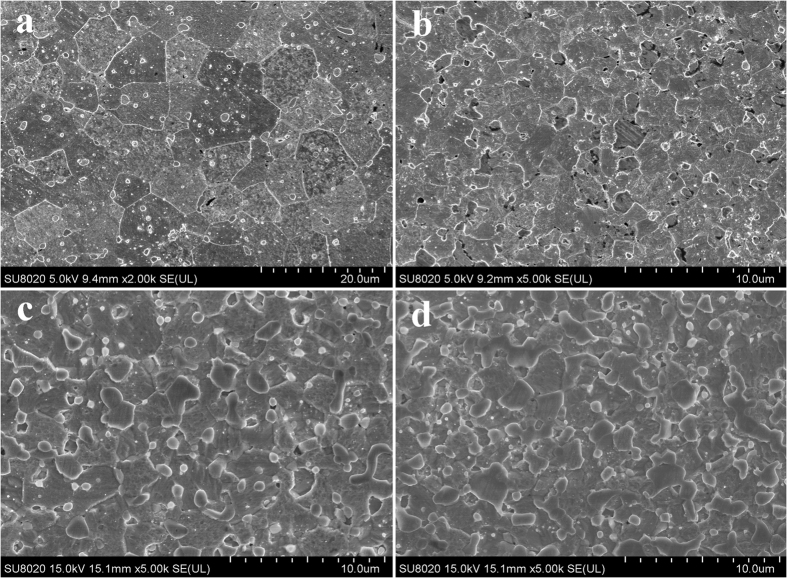
Surface FESEM micrographs of polished and etched (**a**) pure W; (**b**) W-2%Lu; (**c**) W-5%Lu; and (**d**) W-10%Lu.

**Figure 7:**
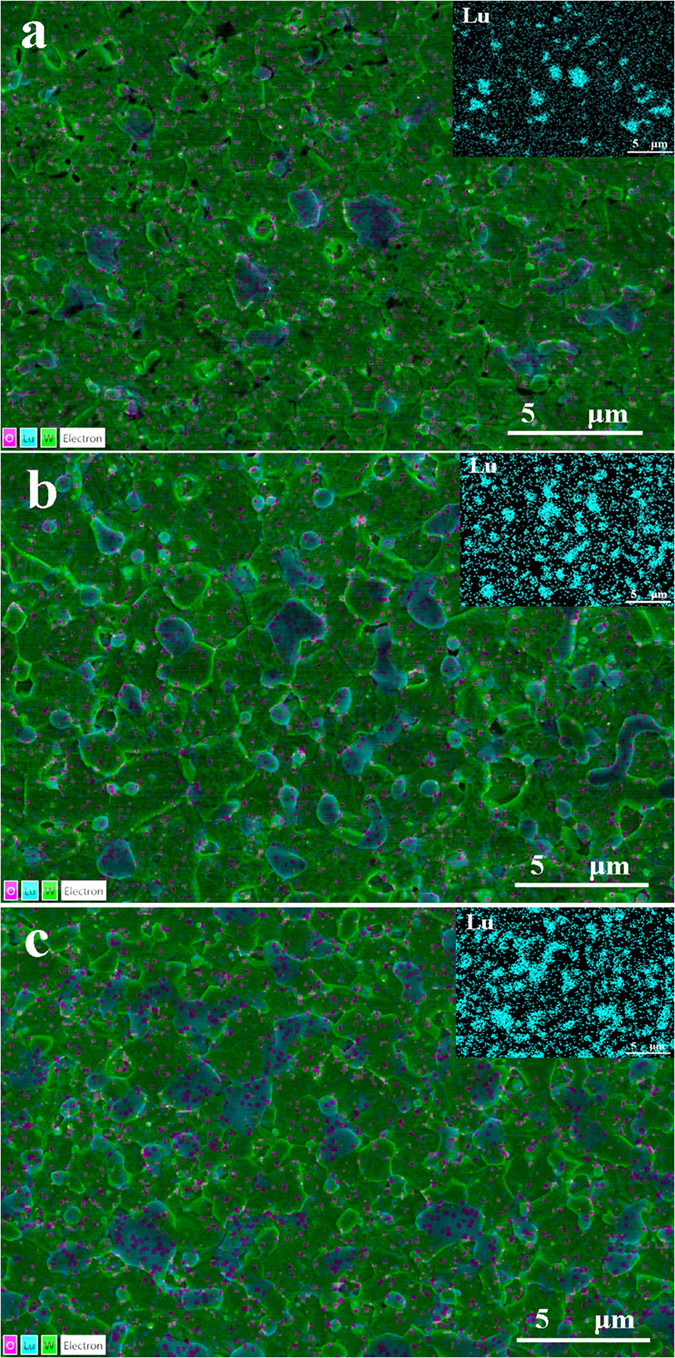
Surface EDS maps of (**a**) W-2%Lu; (**b**) W-5%Lu; and (**c**) W-10%Lu samples. Green, blue, and red regions represent W, Lu, and O elements, respectively. Insets show the distribution of the Lu element.

Figure 6 presents the surface FESEM micrographs of polished and etched samples of the pure W and W-Lu alloys. The images show an apparent decrease in W grain size from 10 μm to 2–3 μm with increasing addition of Lu, indicating an improved W microstructure. The refinement of W grains is attributed to the dispersive particles. Lu resulting from decomposition of LuH_2_ can bind with oxygen to form Lu_2_O_3_ and inhibit the movement of dislocations and grain boundaries to preclude grain growth during the sintering process. EDS maps reveal the distribution of W, Lu and O in the samples. As shown in Fig. 7, the oxide phases that were identified as Lu_2_O_3_ particles in the subsequent section were completely distributed over the W grain boundaries. The amount and size of the dispersed phases clearly increased with increasing Lu content. Excessive amounts of Lu appeared to result in some agglomeration of the second phases in the W-10%Lu alloy, which may cause stress concentration and weaken the W grain boundaries.

**Figure 8:**
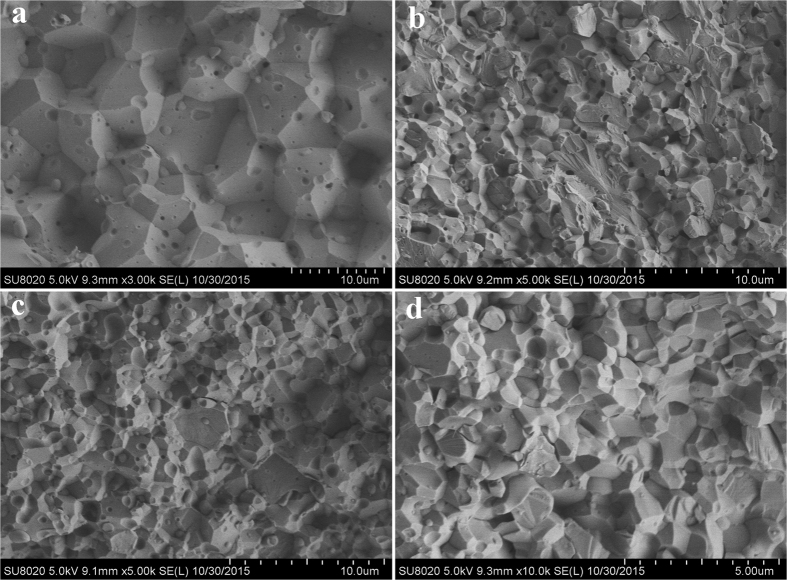
Fracture FESEM micrographs of (**a**) pure W; (**b**) W-2%Lu; (**c**) W-5%Lu; and (**d**) W-10%Lu.

Figure 8 presents the fracture surfaces of the sintered samples. Different fracture mechanisms are observed in the different morphologies among the samples. Pure W exhibits a typical intergranular fracture feature that indicates severe brittleness. However, transgranular and intergranular fractures are observed on the surfaces of the sintered W-Lu samples, particularly on the surface of the W-2%Lu alloy. Figure 8(b) shows several lacerations of W grains on the surface. These lacerations likely originated from the different crack propagation paths changed by the second phase to absorb more energy during the fracture process. The presence of the lacerations and transgranular features indicate improved strength and toughness to some extent. However, the W-10%Lu alloy did not perform well. The distribution and size of the second phase substantially influence the performance of the matrix. Excessive addition of Lu resulted in the accumulation of larger oxides on the grain boundaries, which can serve as the main crack initiation sites, and weakened the W grain boundaries.

**Figure 9:**
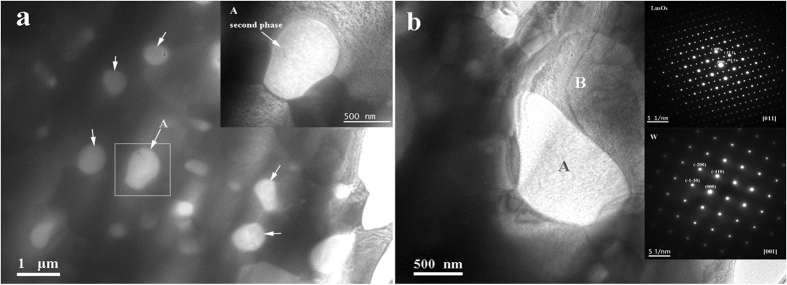
(**a**) TEM micrograph of W-10%Lu alloy and the inset showing the magnified image of the second phase particle A in the grain boundary; (**b**) TEM micrograph of W-10%Lu alloy and insets showing [011] zone axis SAED pattern of the second phase particle on the grain boundary taken from A and [001] zone axis SAED pattern of W matrix taken from B.

TEM analysis was conducted on the W-10%Lu alloy to further characterize its microstructure. The bright-field TEM images of the sintered W-10%Lu alloy are shown in Fig. 9(a,b). As shown in Fig. 9(a), the entire microstructure is composed of dark W grains and bright, dispersedly distributed, submicron-sized second-phase particles (marked with white arrows); the sizes of the W grains and second-phase particles are approximately 1–2 μm and less than 800 nm, respectively. The fine grain size is attributed to the low sintering temperature, the rapid heating rate of SPS, and the pinning effect of dispersive phases on the W grain boundaries. The particles were located entirely in the W grain boundaries and were connected to the matrix, as shown in inset A in Fig. 9(a). The insets in Fig. 9(b) show the selected-area electron diffraction (SAED) pattern of a second-phase particle on the grain boundary (area A) and that of the W matrix (area B). The W phase exhibited a body-centered cubic structure along the [001] zone axis and the second phase was monocrystalline with a body-centered-cubic structure along the [011] zone axis. This result also demonstrates that Lu was oxidized during the sintering process because of its affinity for oxygen impurities in W.

### Irradiation performance: D retention

**Figure 10:**
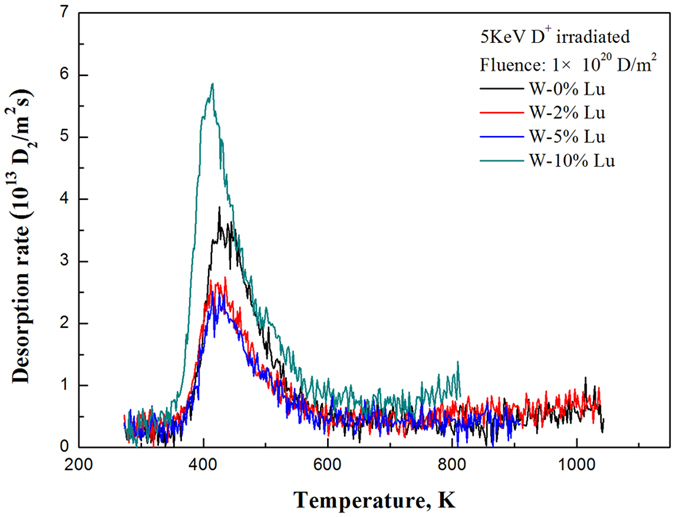
TDS spectra of D_2_ for the pure W and W-(2%, 5%, 10%) Lu samples irradiated with 5 keV D^+^ ions to a fluence of 1 × 10^20^ D/m^2^, as measured during heating from 273 to 1000 K with a fixed heating rate of 1 K/s.

The actual amount of hydrogen retention in W mainly depends on the defect density in the material. Defects such as vacancies, dislocations, clusters, voids, or gas bubbles in the lattice can serve as trapping sites for hydrogen^15^,^20^,^21^. Figure 10 shows the D_2_ desorption spectra of irradiated samples of pure W and W-(2%, 5%, 10%) Lu from 273 K to 1,000 K with a fixed heating rate of 1 K/s. The desorption peaks of the W-(2%, 5%, 10%) Lu samples are basically in the same position and are all shifted toward a slightly lower temperature than the pure W sample. Only one peak was observed in the TDS spectra of all samples and the observed positions were approximately identical (approximately 425 K), indicating that the type of trapping sites in the pure W was similar to that in the W-Lu samples. The D retention in W-2%Lu and W-5%Lu is less than that in pure W and W-10%Lu composites, which is attributable to the decrease in the concentration of trapping sites with the addition of Lu. The finer grains in W-Lu alloys provide more grain boundary areas that serve as defect sinks. That is, the trapping sites for hydrogen decreased. However, the W-10%Lu sample showed comparatively low D^+^ irradiation resistance possibly because of the excessive amount of Lu. The aggregation of D on the interfaces between W and the second phase were easier because of the relatively weak adhesion strength of the agglomerated second phases.

**Figure 11:**
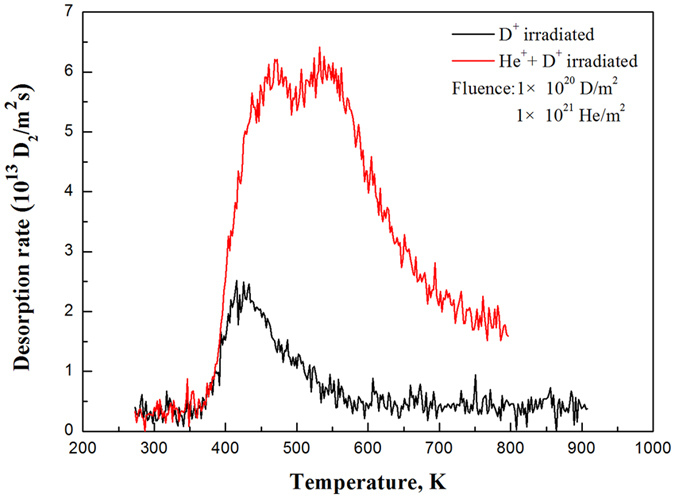
TDS spectra of helium for the W-5%Lu and W-5%Lu samples pre-irradiated with 5 keV He^+^ ions to a fluence of 1 × 10^21^ He/m^2^.

In addition to hydrogen isotopes, PFMs also suffered because of exposure to He particles generated from fusion reactions. The trapping mechanism of D atoms is related to energetic D^+^ and He^+^ irradiation^22^,^23^. The W-5%Lu composites irradiated with He^+^ and D^+^ ions exhibited greater D retention than those subjected to only D^+^ radiation, and two desorption peaks were detected in the TDS spectra (at approximately 470 and 530 K), as shown in Fig. 11. This result demonstrates that the process of pre-irradiation with He^+^ ions dramatically enhanced the retention of hydrogen atoms. The increased D retention and additional peak indicate the increased amount and type of trapping sites in the material. He^+^ irradiation could affect the surface topography of the sample and increase the roughness so that the concentration of the trapping sites is increased dramatically. The effects of He^+^ pre-implantation were consistent with the results reported in previous studies^24^,^25^. The D amount retained in the investigated samples irradiated with D^+^, as measured by TDS technique during heating from 273 to 800 K, is listed in Table 2. The D retention of the W-5%Lu alloy was the lowest, indicating better irradiation resistance.

## Conclusions

The addition of Lu dramatically enhanced the microhardness of the obtained bulk materials from 408.4 Hv to 634.6 Hv, whereas the density initially increased and then decreased with increasing Lu content. SEM, TEM and EDS analyses demonstrated that the submicron-sized second-phase particles, identified as Lu_2_O_3_ particles, were completely distributed over the W grain boundaries and resulted in an effective grain refinement. Lu generated by decomposition of LuH_2_ bound with oxygen in W to form Lu_2_O_3_ particles because of the strong affinity of Lu for oxygen impurities. The second phases pinned the dislocations and grain boundaries to preclude grain growth during the sintering process. The amount and size of the dispersive phases clearly increased with increasing Lu content. Excessive addition of Lu resulted in some agglomeration of the second phases on the grain boundaries, which can concentrate stress and weaken the W grain boundaries. Transgranular and intergranular fracture modes were observed on the fracture surface of the sintered W-Lu samples, indicating some improvement of strength and toughness. The W-(2%, 5%) Lu alloys exhibited greater resistance than pure W to D^+^ radiation, whereas W-10%Lu exhibited the highest D retention and a comparatively low irradiation performance. The process of pre-irradiation with He^+^ ions promoted the retention of D, possibly because of the increase in the amount and type of trapping sites in the material after He^+^ irradiation. The amount and distribution of Lu substantially affected the properties of W. W-5%Lu exhibited the best overall performance among the investigated samples.

## Additional Information

**How to cite this article**: Luo, L. *et al*. Microstructure and performance of rare earth element-strengthened plasma-facing tungsten material. *Sci. Rep.* **6**, 32701; doi: 10.1038/srep32701 (2016).

## Figures and Tables

**Table 1:** Density and relative density of W and W-Lu alloys sintered by SPS.

Material	ρ_theo_ (g/cm^3^)	ρ_expe_ (g/cm^3^)	Relative density (%)
Pure W	19.25	17.33	90.0%
W-2%Lu	19.06	17.41	91.3%
W-5%Lu	18.78	17.78	94.7%
W-10%Lu	18.31	17.01	92.9%

**Table 2:** Amount of deuterium retained in the investigated samples irradiated by D^+^ and He^+^+D^+^ from 273 K to 800 K.

Sample	Amount of retained deuterium (D/m^2^)
W	5.15 × 10^15^
W-2%Lu	4.41 × 10^15^
W-5%Lu (D^+^-only)	4.03 × 10^15^
W-5%Lu (He^+^+D^+^)	1.64 × 10^16^
W-10%Lu	8.09 × 10^15^
